# Using Time Trade-Off Methods to Elicit Short-Term Utilities Associated with Treatments for Bulbar Urethral Stricture

**DOI:** 10.1007/s41669-019-0133-4

**Published:** 2019-06-25

**Authors:** Jing Shen, Matthew Breckons, Luke Vale, Robert Pickard, for the OPEN trial investigators

**Affiliations:** 1 Health Economics Group, Institute of Health and Society, Newcastle University, Baddiley-Clark Building, Richardson Road, Newcastle upon Tyne, Tyne and Wear NE2 4AX UK; 2 Institute of Cellular Medicine, Newcastle University, Newcastle upon Tyne, UK

## Abstract

**Background:**

Recurrent urethral stricture is usually treated with either open urethroplasty or endoscopic urethrotomy. Both of the procedures cause short-term utility loss, which may not be captured by standard utility questionnaires due to the challenges of completing a standard instrument at the time of an acute episode of short duration, especially within a clinical trial setting. We propose to use time trade-off (TTO) methods to estimate these short-term utility losses.

**Objective:**

The aim was to compare the use of two alternative TTO methods to elicit patients’ short-term utilities following surgical treatments for recurrent urethral stricture.

**Method:**

Two variants of TTO (chained and conventional) were used. Six health profiles were developed—three for each procedure. Forty participants took part, with 20 randomly allocated to each TTO method.

**Results:**

Thirty-eight participants provided usable data for analysis. Estimated utility values decreased as the severity of the health profiles increased. There was no evidence that utility values differed between elicitation methods or procedures for mild {ranging from 0.79 (standard deviation [SD] 0.17) to 0.83 [SD 0.20]} and moderate (ranging from 0.54 [SD 0.24] to 0.67 [SD 0.21]) health states, although they appeared to differ for severe health states (ranging from 0.29 [SD 0.20] to 0.56 [SD 0.24]).

**Conclusion:**

The study demonstrates the feasibility and value of eliciting patients’ short-term utilities. Given the small sample size, the study findings are tentative. Further research with a larger sample size is needed to determine the appropriate TTO method to use and how the elicited utilities can be used in combination with standard cost-utility assessments to aid decision making.

**Electronic supplementary material:**

The online version of this article (10.1007/s41669-019-0133-4) contains supplementary material, which is available to authorized users.

## Key Points for Decision Makers

**Table:** 

This study makes the first attempt to compare the use of two alternative time trade-off methods to elicit patients’ short-term utilities following surgical treatments.
The study shows the feasibility and value of eliciting patients’ utilities as part of a clinical trial when routine data collection was not able to capture all utilities.
The choice of elicitation method may depend on the severity of health states to detect any meaningful difference.

## Introduction

There is a growing emphasis on measuring and evaluating patients’ health-related quality-of-life (HRQoL), which is converted into utility values used in economic evaluations. As an important outcome, it is key to ensure all utilities are captured in the analysis to ensure a robust and accurate comparison between treatment strategies. This is sometimes difficult to achieve due to a combination of the fixed frequency of HRQoL data collection, unpredictable recurrence of disease-specific events that impact on patients’ HRQoL, and the unfeasibility of collecting HRQoL data immediately after such events. This is the case in the OPEN (Open Urethroplasty versus Endoscopic Urethrotomy) study [1], which was a randomised controlled trial comparing two treatment strategies (open urethroplasty versus endoscopic urethrotomy) for men with recurrent urethral stricture. Urethral stricture is a narrowing of the urethra caused by scarring after injury or infection and is the most common cause of difficulty passing urine in younger and middle-aged men [2]. Endoscopic urethrotomy, a procedure in which the stricture is divided using an instrument passed along the urethra, is commonly performed for recurrent bulbar stricture because it is minimally invasive, does not require specialist surgical expertise, and has a short period of urethral catheterisation and recovery. However, further recurrence is likely [3]. Open urethroplasty, where the urethra is surgically reconstructed through an incision in the perineum, is more invasive, requires specialist expertise and a longer period of catheterisation and may be complicated by wound pain and infection. It does, however, offer the prospect of long-term cure without the need for further interventions [4, 5]. The current decision-making process in the UK National Health Service (NHS) is influenced by availability of local expertise, clinician guidance as well as patient preferences.

The OPEN trial aimed to compare the clinical- and cost-effectiveness of alternative treatments for recurrent urethral stricture in men and resolve uncertainty as to whether men with a recurrent urethral stricture are best treated by endoscopic urethrotomy or open urethroplasty [1]. The health economic component of the OPEN study measured the effects of the procedures in terms of quality-adjusted life years (QALYs) derived from the EQ-5D-5L questionnaire administrated at baseline and then at a 6-monthly interval. Because of the invasiveness of the procedures and their associated side effects, participants’ HRQoL was likely to temporarily deteriorate post-treatment, but this would not be captured by completion of a later scheduled EQ-5D as respondents were asked about health on the day the questionnaire was completed. Furthermore, the recurrence of urethral stricture is unpredictable.

This proves a challenge, as data collection at fixed time periods has the potential to miss short-term but frequent changes to HRQoL, and within a large-scale, multi-centre study, it is not feasible to collect data using an individualised follow-up schedule. Furthermore, it is usually not acceptable in terms of participant burden to ask participants to complete an HRQoL questionnaire at the time of the event occurrence when they are unwell. Therefore, a time trade-off (TTO) exercise was conducted to elicit the short-term utilities that would otherwise be missed.

TTO is one of a number of methods to measure preferences for temporary health states for cost-utility analysis [6], eliciting the impact of impaired health on individuals’ quality of life by asking participants to state preferences between quality and quantity of life in hypothetical scenarios. The impact is measured in terms of utility values that usually fall between 0 and 1, where 0 is equated to ‘being dead’ and 1 ‘being in perfect health’, though negative values are possible for health states considered worse than death [7]. The TTO method has mostly been used to elicit utility values for chronic health states where participants typically remain in the impaired health state for 10 years or more [8], and we have termed this as a ‘conventional TTO’. However, the two surgical procedures in the OPEN study were likely to have a short-term impact on patients’ HRQoL over days or weeks post-operatively before the patients returned to usual health. In these circumstances, a conventional TTO exercise may become less responsive [9, 10]. This is because the exercise offers an unrealistic choice between an impaired health state for a fixed duration and a perfect health state for a shorter duration of time, both followed by death. Attempts to remedy this problem have involved using an intermediate health state rather than directly comparing the temporary health state to perfect health and death, and this method is referred to as the ‘chained TTO’ [11]. However, little research has been done on the performance of conventional and chained TTO methods in eliciting short-term utility values. As an exploratory first attempt, the aims of this TTO study were, therefore:

- To assess the feasibility of eliciting short-term utilities for health states resulting from treatments investigated in a clinical trial.
- To tentatively compare utility values elicited using conventional and chained TTO methods.

## Methods

### Participants

The TTO study took place in parallel with the OPEN trial. The TTO participants were recruited from those who were eligible for the OPEN trial: males aged 16 years or over, with a stricture located predominantly in the bulbar urethra, who had undergone at least one previous intervention for bulbar urethral stricture; clinical and patient agreement that further intervention was required; patients suitable for necessary anaesthesia who were willing to undergo up to 2 weeks of catheterisation and provided written consent for study participation. All screened eligible OPEN trial patients were asked to indicate whether they would be interested in participating in an interview study regardless of their decision about whether to participate in the main OPEN trial. Those who expressed interest were posted a TTO Study Information Pack containing a response slip and pre-paid envelope. Upon receipt of an affirmative response slip, a researcher contacted respondents to answer any further questions and arrange a time and place of the participant’s choosing to conduct the TTO interview.

### TTO Materials

Three health state profiles were created for each procedure, representing ‘mild’, ‘moderate’ and ‘severe’ health states based on the severity of side effects following each procedure. The profiles were developed based on consultation with clinicians (urologists from the OPEN trial main site) and a patient co-investigator, as well as findings from qualitative interviews conducted in the pilot phase of the trial where participants provided a personal account of their symptoms and the impact on their quality of life [12]. The time horizon chosen for the health states was based on the shortest time length during which most of the side effects would occur—14 days. The urethrotomy profiles focused on differing severities of urinary symptoms: discomfort from the catheter, bleeding on urination, urinary tract infection and erectile dysfunction. While the nature of the symptoms was similar for each level of severity, these were differentiated by descriptors (e.g. brief/serious) and the addition of more serious side effects such as infections. The urethroplasty profiles were nearly identical but incorporated the additional symptoms from the graft donor site in the mouth and perineal wound. Profiles are presented in Appendix 1 [see the electronic supplementary material (ESM)]. The anchor state [9, 10] for use in the chained TTO version described an injured state in which basic tasks could be carried out but usual activities such as work and socialising were not possible and pain was constant (Appendix 2; see the ESM). Piloting of the health states and the anchor state [13] ensured that the anchor state was considered worse than the health states but better than death.

As a warm-up task, a set of practice profiles was chosen from the EQ-5D-3L profiles to allow participants to become familiar with the TTO task prior to valuing the study health states. Using practice profiles is a standard practice for TTO studies [14, 15] to improve participants’ understanding of the TTO exercise and thus improve data quality. Further, in our study, we also asked each participant to value an additional set of three different EQ-5D-3L profiles after evaluating the study-specific health states. The purpose of evaluating these extra profiles was to provide further comparisons between utility values derived from the conventional and chained TTO methods as those additional EQ-5D-3L profiles (11211, 12222, 23321) have directly elicited tariffs values from the UK population [15].

An A3-sized decision board was constructed to assist in the TTO interviews, and all of the health profiles were printed on coloured and laminated A6-size cards, using a different colour for each profile. The TTO materials and process were extensively tested and piloted as described elsewhere [13].

### TTO Interviews

All interviews were conducted face to face by an interviewer trained in TTO methods (JS, MB and the other health economists listed in the acknowledgements). Interviews were most frequently conducted in participants’ own homes, and written consent was taken prior to commencing the interview. Following the practice task, participants were asked to rank the six health state profiles from best to worst, after which the profile cards were shuffled by the interviewer and then evaluated by the participants in the TTO exercise. Following valuation of the six profiles, participants were asked to value the three additional EQ-5D profiles, after which those in the chained group were given a practice task followed by valuation of the anchor state using the conventional TTO method (i.e. everyone valued 12 profiles in total, with those in the chained group being given a further two profiles). The iteration procedure resembled a ‘ping-pong’ approach [16], i.e. the length of the state being valued was alternated between 14 days and 1 day, 13 days and 2 days, etc. until the participant identified a time period where they were indifferent to the two states. While the board displayed only whole numbers of days, participants were informed that they could select a proportion of a day if they wished given the short timeframe.

### Data Analysis

Information on the sociodemographic characteristics of the participants was collected and used in the analysis, including age, marital status, income, education, employment status, physical activity level and urban/rural residency. The latter two variables were included because it was assumed that participants’ usual physical activity level and the location of their residence would have an impact on how they valued those health states that would impact on their mobility.

Utility values were calculated as follows:

*Conventional TTO*: the Utility value for each health state (*h*_*i*_) was calculated using the formula *h*_*i*_ = *x*/*t,* where *x* is the time point at which a participant is indifferent to spending *x* days in perfect health and *t* days in the health state (fixed at 14 days).

*Chained TTO*: The utility values for each health state (*h*_*i*_) and the anchor state (*h*_*j*_) were calculated using the following formulas. In the first formula, *x*_1_ is the time point at which the participant is indifferent to spending *x*_1_ days in the anchor state and *t* days (14) in the health state. The second formula calculates the utility value for the anchor state (*h*_*j*_) using the conventional method where *x*_2_ is the time point at which the participant is indifferent to being in the anchor state or *t* days (14) in perfect health:

$$h_{i} = 1 - \left( {1 - h_{j} } \right)\frac{{x_{1} }}{t}$$

$$h_{j} = \frac{{x_{2} }}{t},$$

The combined formula for calculating the utility value for each health state is then:

$$h_{i} = 1 - \left( {1 - \frac{{X_{2} }}{t}} \right)\frac{{X_{1} }}{t}.$$

Tobit regressions of the elicited TTO values were performed to derive predicted utility values for each health state controlling for sociodemographic characteristics. An additional control variable was created based on the consistency between the utility values derived and how each participant ranked those profiles prior to the TTO exercise to indicate data quality. Estimates of predicted utility values for each procedure and each elicitation method were then compared using *t* tests. Stata (version 14; StataCorp LP) was used to analyse the data. The regression equation is described as follows, where *U* is the elicited utility value, *x*_*i*_ represents the set of explanatory variables, with *α* and *ɛ* as the constant and error term, respectively. This is performed for each of the six health profiles evaluated and the three additional EQ-5D profiles:

$$U = \alpha + \beta_{i} x_{i} + \varepsilon .$$

Separate models were performed for each health profile instead of being combined in a panel framework for two reasons. Firstly, the health state profiles were designed and valued as a whole for the needs of this study rather than selected based on systematic variations in the dimensions; therefore, dummy variables for the dimensions were not available to be included in the regressions, without which the health states utility values cannot be estimated in a panel framework. Secondly, running separate models allowed for the possibility that the impact of one or more of the control variables may not be uniform across the range of mild, moderate and severe health states. This is especially important given the small sample size. Detailed regression results are presented in Appendix 4 (see the ESM).

## Results

A total of 40 participants were recruited to the study, with 20 allocated randomly to each TTO method. Two participants from the chained TTO group were excluded from the analysis due to missing data (did not wish to value the anchor state) and non-trading (did not want to trade the anchor state with death), respectively. Of those included in the analysis, the average age was 54 years. The majority of the participants were married (84%), 34% had degree level and above education, 39% had a household income above £36,400, 55% were employed and 29% were retired. Most of the participants (71%) lived in the urban area. Levels of physical activity were reported at 29, 47 and 24% for high, median and low, respectively. Full summary statistics are given in Appendix 3 (see the ESM).

Overall, the mean estimated utility values consistently decreased with increasing severity of health states within each procedure for both TTO methods, demonstrating face validity of the elicited values.

Table 1 compares each of the predicted health state utility values between the two procedures. For both types of TTO methods, utilities were lower for urethroplasty, but the difference was only significant for the severe health states as shown by the *t* statistics and their associated *P* values at a significance of above 95%. For all the other health states, there is no evidence of a difference between the two procedures.

**Table 1:** Comparing utility values between the surgical procedures

Types of TTO methods	Health states severity	Urethrotomy, mean (SD)	Urethroplasty, mean (SD)	Mean difference	95% CI of mean difference	*P* value (*t* statistics)
Conventional (*n* = 20)	Mild	0.81 (0.19)	0.79 (0.17)	0.01	− 0.04 to 0.07	0.59 (0.51)
	Moderate	0.58 (0.30)	0.54 (0.24)	0.04	− 0.07 to 0.15	0.47 (0.72)
	Severe	0.56 (0.24)	0.39 (0.27)	0.17***	0.08 to 0.27	0.00 (3.81)
Chained (*n* = 18)	Mild	0.83 (0.18)	0.83 (0.20)	0.01	− 0.03 to 0.04	0.77 (0.38)
	Moderate	0.67 (0.21)	0.62 (0.15)	0.05	− 0.08 to 0.18	0.43 (0.81)
	Severe	0.44 (0.19)	0.29 (0.20)	0.15**	0.02 to 0.28	0.04 (2.36)

*CI* confidence interval, *SD* standard deviation, *TTO* time trade-off

***P < 0.01, **P < 0.05

Table 2 compares each of the predicted health state utility values between the two TTO methods. For the mild and moderate health states, conventional TTO appeared to generate lower utility values than chained TTO, whereas for the severe health states the opposite is true. However, *t* test results suggest that the differences are not statistically significant except for the severe urethrotomy health state, where the *t* statistic is large enough to have a *P* value at 90%. However, this difference may still be due to chance given the small sample size, as the confidence intervals for the mean differences included 0.

**Table 2:** Comparing utility values between the TTO methods

Surgical procedures	Health states severity	Conventional TTO, mean (SD) (*n* = 20)	Chained TTO, mean (SD) (*n* = 18)	Mean difference	95% CI of mean difference	*P* value (*t* statistics)
Urethrotomy	Mild	0.81 (0.19)	0.83 (0.18)	− 0.03	− 0.15 to 0.10	0.65 (− 0.44)
	Moderate	0.58 (0.30)	0.67 (0.21)	− 0.09	− 0.26 to 0.08	0.28 (− 1.04)
	Severe	0.56 (0.24)	0.44 (0.19)	0.12*	− 0.02 to 0.26	0.09 (1.73)
Urethroplasty	Mild	0.79 (0.17)	0.83 (0.20)	− 0.03	− 0.15 to 0.09	0.52 (−0.57)
	Moderate	0.54 (0.24)	0.62 (0.15)	− 0.08	− 0.21 to 0.05	0.23 (− 1.22)
	Severe	0.39 (0.27)	0.29 (0.20)	0.10	− 0.06 to 0.25	0.22 (1.27)

*CI* confidence interval, *SD* standard deviation, *TTO* time trade-off

*P < 0.1

Table 3 compares the predicted utility values for the additional EQ-5D-3L profiles evaluated at the end of the TTO exercise between the two TTO methods. Similar trends were observed where conventional and chained TTO methods appeared to generate similar utility values for mild and moderate health states, but for the most severe health state, conventional TTO resulted in a lower utility value than chained TTO, and the difference is statistically significant at 95%. Additionally, comparison with the EQ-5D-3L UK reference tariffs showed that for the mild profile (11211), there was no evidence of a difference between the predicted utility values from the conventional method and the national tariff. There were notable differences between all other predicted utility values and the national tariff, and the utility values generated in the present study are significantly higher than the national tariff.

**Table 3:** Comparing utility values elicited based on EQ-5D-3L profiles

Health states (national tariff)	Conventional TTO, mean (SD) (*n* = 20)	Chained TTO, mean (SD) (*n* = 18)	Mean difference	95% CI of mean difference	*P* value (comparison between TTO methods) (*t* statistics)	*P* value (conventional TTO compared to national tariff) (*t* statistics)	*P* value (chained TTO compared to national tariff) (*t* statistics)
EQ-5D profile 11211 (0.869)	0.89 (0.11)	0.93 (0.09)	− 0.04	− 0.11 to 0.02	0.18 (− 1.38)	0.41 (0.84)	0.01*** (3.20)
EQ-5D profile 12222 (0.551)	0.79 (0.17)	0.78 (0.17)	0.01	− 0.10 to 0.12	0.83 (0.21)	0.00*** (6.34)	0.00*** (5.61)
EQ-5D profile 23321 (0.147)	0.59 (0.24)	0.77 (0.17)	− 0.17**	− 0.31 to −0.03	0.02** (− 2.54)	0.00*** (8.29)	0.00*** (15.21)

*CI* confidence interval, *SD* standard deviation, *TTO* time trade-off

***P < 0.01, **P < 0.05

The results appear to suggest that for mild or moderate health states, the utility values generated from both TTO methods appear to be robust, but the utility values are highly influenced by the TTO method chosen when more severe health states are considered.

## Discussion

Our review of the published literature suggests this is the first study to use TTO to capture short-term utilities associated with interventions investigated in a clinical trial and one of a small number to use the chained TTO method [17]. Given the short-term nature of the health states being evaluated where conventional TTO was suggested to be subject to bias, this study also aimed to explore the performances of two TTO methods in eliciting short-term utilities. However, as this was conceived as an exploratory first attempt, there was no intention to provide definitive estimates on the short-term utilities; hence, a small sample size was used to fit within the scope of the clinical study. Nevertheless, this study provides evidence of the acceptability and feasibility of a TTO study conducted alongside a clinical trial, as well as some suggestions regarding justifications for using each TTO method.

While TTO studies often focus on health states over a period of several years [18, 19] or even ‘the rest of life’ [20], this study attempted to elicit utilities over a very short time period immediately following the treatment procedures. The estimated utility values associated with each procedure decrease as the health states become worse, suggesting these estimations had face validity. The high rate of useable data indicates that conducting a TTO exercise alongside a clinical trial is both acceptable and feasible.

Comparison between the two procedures showed that although most differences were not statistically significant, lower utility values were associated with the urethroplasty-related health states, which was expected as urethroplasty was the more invasive procedure. This finding suggests a greater decrement in HRQoL would be expected immediately following the urethroplasty procedure. This could be because participants were particularly averse to one or more of the symptoms described in the urethroplasty health states. However, we cannot rule out a possible type 1 error due to the small sample size. Additionally, there is evidence suggesting TTO methods suffer from inherent bias, but findings have been mixed, with one study suggesting biases were associated with both the chained and conventional TTO methods [10], while another [21] showed that the chained method avoids biases observed using the conventional TTO.

Comparing the two TTO methods, there is no evidence that the estimated utility values differ for mild and moderate health state profiles, but values did diverge when severe health states were considered. This pattern was observed for both the disease-specific health states and the additional EQ-5D-3L health states valued at the end of the interview, although the chained TTO produced lower utility for the worst disease-specific health state but higher utility for the worst EQ-5D-3L health state, which is an interesting and unexpected finding that requires further investigation with a sufficient sample size. Given the small sample size in our study, we cannot rule out this finding is simply due to chance.

A further unexpected finding to note is that utilities for 12222 and 23321 EQ-5D-3L health states estimated in the present study were significantly higher than the national tariff. While given the small sample size we do not want to be overly reliant on the statistical significance, we may speculate on reasons for this interesting finding: these EQ-5D-3L profiles were evaluated at the end of the TTO interview, and following the valuation of some very severe health states, participants may find those EQ-5D-3L profiles less severe in comparison; another possible explanation is that those EQ-5D-3L profiles were valued by a patient population who had experienced some very distressing and troublesome health conditions, and therefore, they may consider the EQ-5D-3L profiles less severe than the general population whom the national tariff was based on. This may raise the question on the role a patient’s own medical history plays in their valuation of health states, which has been explored previously. For example, Jansen et al. [9] examined the stability of preferences before, during and after treatment. We were not able to examine the patient valuations against their treatment timeframe in the study, and future study design should aim to enable this for such investigation.

This study has a number of limitations. The most notable one is the small sample size, which means a wider standard deviation for all the study estimates, and we cannot rule out any finding being simply due to chance. The use of randomisation to determine which TTO method a respondent received theoretically helped ensure the two groups were balanced and comparable. In practice, however, the small sample size means this may not be enough to ensure a balanced sample between the two groups. Additionally, heteroscedasticity among study participants is inevitable with a small sample. To remedy those, we adjusted for observable individual characteristics to estimate the utility values, which would help reduce potential bias in estimations; however, the regressions’ degree of freedom was sacrificed as a result of the added number of explanatory variables and small sample size, and this reduces the precision of the estimates. Due to the limited scope of the present study, we were unable to test the external validity of the utility estimates, and given the small sample size, the study sample may not be representative of the patient population.

We have only been able to examine statistical significance when comparing the estimated results. A more meaningful investigation would be to examine whether these differences were relevant from a clinical perspective by considering estimated difference against the minimally important difference (MID) [22] for a TTO approach for the health states in question. For utility values associated with EQ-5D-3L (which was derived from responses to a set of conventional TTO questions), the MIDs have been variously estimated: a study examining MIDs from patients with a range of health conditions found a mean MID of 0.074 [23], and another study examining MIDs for cancer patients reported a mean MID of 0.09 [24]. Only the moderate and severe health states when comparing between TTO methods and severe health states when comparing between treatments in our study appear to exceed the MIDs found in the literature. This may indicate that a meaningful difference in HRQoL is only important when the health states are more distressing or troublesome. Correspondingly, for mild health states, the choice of TTO method may not be crucial because the differences are not important to patients or clinicians. However, for more severe health states, we need to carefully consider which TTO method to use. The issue of what the MID would be in this context is clearly important to explore, with the input from both patients and clinicians. Recent guidance has suggested alternative methods to do this [25, 26]. Further work could also be designed to evaluate clinicians’ as well as patients’ preferences over the health states in question, to better understand how the perceived impact from treatments may vary between patients and clinicians and aid shared decision making for patient-centred care [18].

Understanding of the short-term impact of the procedures would offer valuable information from a policy-making perspective as well as improve patient information on treatment choices. Further work combining the TTO data with other observations such as relative incidence of side effects and recurrence rates may help to support decision making in the NHS regarding the choice between urethrotomy and urethroplasty.

The chained TTO may have a stronger theoretical base [11], and other studies using chained TTOs have suggested that this is a responsive method for eliciting preferences [9, 27, 28]. However, if the value of using chained TTO is not reflected in improving the accuracy of estimated utility values, it might not justify the additional burden and complexity of replacing the conventional TTO in the instances of short-term health states valuation. Conventional TTO, on the other hand, is easier to design, administer and complete. However, further research with a larger sample size is needed to establish the justification in the selection between the two TTO methods. There is also a need to investigate consistency and reliability within the same participant [10].

## Conclusion

Using TTO to elicit utilities for short-term health states alongside a clinical trial has proven to be feasible and acceptable. While the study finding is preliminary, it suggests that undergoing urethroplasty or urethrotomy is likely to result in a decrement to HRQoL immediately post-operation, with the former resulting in likely higher utility losses. The tendency of men with urethral stricture to require repeated treatments increases the clinical significance of this decrement, which should be incorporated into the QALY-based cost-effectiveness assessment of each treatment strategy in the longer term. This study also explored the rationale for applying the more complex chained TTO method for short-term health state valuations. The indicative results suggest that chained TTO overall does not result in significantly different estimates from conventional TTO when the health states are mild or moderate. In the context of severe health states, there may be justification for the use of chained TTO. Further research with a sample size calculation and direct comparison with an alternative method of deriving utilities is needed to establish justification for the use of the chained TTO method if results are proven to be more accurate and robust. Future studies may also explore the potential to incorporate the TTO estimates in the cost-effective analysis, with consideration given to achieving sufficient sample size and the selection of the TTO participants (public, patients or clinicians) for the perspective of evaluation.

## Electronic supplementary material

Below is the link to the electronic supplementary material.

Supplementary material 1 (DOCX 33 kb)
