# Supplementary material for: Using Time Trade-Off Methods to Elicit Short-Term Utilities Associated with Treatments for Bulbar Urethral Stricture
Source: Pharmacoecon Open. 2019 Jun 25;3(4):551–8. doi: 10.1007/s41669-019-0133-4 (PMC6861395; doi:10.1007/s41669-019-0133-4)
Supplement: Supplementary file 1 — Supplementary material 1 (DOCX 33 kb) [file 41669_2019_133_MOESM1_ESM.docx]

**Appendices**

**Appendix 1. Health States for urethrotomy and urethoplasty**

**Control intervention urethrotomy health state profiles**

Urethrotomy - Mild

- Discomfort in the penis and bladder from using a catheter for a few days
- Brief discomfort on passing urine after the catheter is removed
- A few drops of blood after you have finished passing urine
- Mild urinary tract infection giving you mild fever-like symptoms

Urethrotomy - Moderate

Urethrotomy - Severe

- Discomfort in the penis and bladder from using a catheter
- Severe urethral bleeding which requires you to have a telescopic examination under anaesthetic
- Serious urinary tract infection which makes you feel ill and requires you to stay in hospital overnight for antibiotics from an IV drip
- Severe pain in the penis and bladder area requiring you to take regular painkillers
- Difficulty getting and maintaining a penile erection for sex
- Discomfort in the penis and bladder from using a catheter for a few days
- Discomfort on passing urine after the catheter is removed
- Moderate urethral bleeding which requires you to keep the catheter in longer or have a telescopic examination under anaesthetic
- Serious urinary tract infection which makes you feel ill and requires you to stay in hospital overnight for antibiotics from an IV drip

**Experimental intervention urethroplasty health state profiles**

Urethroplasty – Mild

- Discomfort in the penis and bladder from using a catheter
- Mild mouth pain or discomfort when you eat or drink
- Mild urinary tract infection giving you mild fever-like symptoms
- Mild swelling and wound pain in the area between the testes and back passage

Urethroplasty – Moderate

- Discomfort in the penis and bladder from using a catheter
- Moderate and constant mouth pain and scarring in the mouth needing regular painkillers
- Serious urinary tract and wound infection which makes you feel ill and requires you to stay in hospital overnight for antibiotics from an IV drip
- Moderate wound pain in the area between the testes and back passage needing regular painkillers

Urethroplasty - Severe

- - - - Discomfort in the penis and bladder from using a catheter
      - Severe and constant mouth pain and scarring in the mouth needing regular painkillers
      - Serious urinary tract and wound infection which makes you feel ill and requires you to stay in hospital overnight for antibiotics from an IV drip
      - Severe wound pain in the area between the testes and back passage needing regular painkillers
      - Leakage of urine from the area between the testes and back passage requiring you to wear incontinence pads
      - Difficulty getting and maintaining a penile erection for sex

**Appendix 2. Anchor State**

| You have recently been injured and as a result of the injury:  You are able to do basic tasks (e.g. washing, feeding and communicating) but you have problems walking about  You have extreme pain and discomfort. No medication can completely alleviate the pain  You cannot take part in usual activities (e.g. work, social activities and exercise) |
| --- |

**Appendix 3. Summary statistics of sociodemographic factors used in the regression**

| **Sociodemographic factors** | **Mean (SD)**  **/Percentage** | **n** |
| --- | --- | --- |
| Age (SD) | 54 (14) | 38 |
| Marital status (%) |  |  |
| Married/Co-habitation | 15.79 | 6 |
| Single/ Divorced/Widowed | 84.21 | 32 |
| Household income (%) |  |  |
| <£15599/undisclosed | 28.95 | 11 |
| Between £15599 and £36399 | 31.58 | 12 |
| >£36400 | 39.47 | 15 |
| Education level (%) |  |  |
| Degree level or above | 34.21 | 13 |
| Higher education below a degree/ GCE A-Level or AS-Level / NVQ level 3/ GCSE grade A*-C / GCE O-Level / NVQ level 2 | 44.74 | 17 |
| GCSE grade D-G / CSE / NVQ level 1/ Foreign qualifications / other/ No formal qualifications | 21.05 | 8 |
| Employment status (%) |  |  |
| Employed Full-Time/ Self-Employed | 55.26 | 21 |
| Retired | 28.95 | 11 |
| Employed Part-Time/Unemployed | 15.79 | 6 |
| Physical activity level (%) |  |  |
| Low | 23.68 | 9 |
| Median | 47.37 | 18 |
| High | 28.95 | 11 |
| Urban or rural residency (%) |  |  |
| Rural area (Village/Hamlet/Isolated Dwelling) | 28.95 | 11 |
| Urban area (City/Town) | 71.05 | 27 |

**Appendix 4. Regression results**

**Regression results for utilities elicited using conventional TTO method**

| **Covariates** | **Profile 1** | **Profile 2** | **Profile 3** | **Profile 4** | **Profile 5** | **Profile 6** |
| --- | --- | --- | --- | --- | --- | --- |
| **Age** | 0.001(0.004) | 0.013**(0.005) | 0.008**(0.003) | 0.006(0.004) | -0.002(0.006) | 0.015**(0.006) |
| **Marital status** |  |  |  |  |  |  |
| Single/ Divorced/Widowed (reference) | - | - | - | - | - | - |
| Married/Co-habitation | 0.258*(0.116) | 0.199(0.131) | -0.253**(0.087) | 0.051(0.126) | 0.210(0.166) | 0.132(0.149) |
| **Household income** |  |  |  |  |  |  |
| <£15599/undisclosed (reference) | - | - | - | - | - | - |
| Between £15599 and £36399 | -0.187(0.151) | -0.014(0.130) | -0.278**(0.087) | -0.151(0.134) | 0.118(0.168) | 0.207(0.168) |
| >£36400 | -0.107(0.177) | 0.059(0.151) | -0.022(0.102) | 0.002(0.166) | 0.494**(0.201) | 0.519**(0.187) |
| **Education level** |  |  |  |  |  |  |
| GCSE grade D-G / CSE / NVQ level 1/ Foreign qualifications / other/ No formal qualifications (reference) | - | - | - | - | - | - |
| Higher education below a degree/ GCE A-Level or AS-Level / NVQ level 3/ GCSE grade A*-C / GCE O-Level / NVQ level 2 | 0.217(0.132) | 0.171(0.118) | 0.202**(0.080) | 0.006(0.114) | 0.285*(0.152) | 0.097(0.141) |
| Degree level or above | -0.003(0.147) | -0.138(0.149) | -0.107(0.100) | -0.074(0.145) | -0.465**(0.191) | -0.563**(0.213) |
| **Employment status** |  |  |  |  |  |  |
| Employed Part-Time/Unemployed (reference) | - | - | - | - | - | - |
| Retired | -0.030(0.161) | -0.316*(0.158) | -0.237*(0.104) | -0.226(0.152) | -0.307(0.198) | -0.778***(0.225) |
| Employed Full-Time/Self-Employed | 0.034(0.166) | -0.002(0.152) | -0.069(0.099) | 0.103(0.161) | -0.526**(0.194) | -0.400*(0.191) |
| **Physical activity level** |  |  |  |  |  |  |
| Low (reference) | - | - | - | - | - | - |
| Median | 0.081(0.117) | -0.146(0.128) | -0.013(0.086) | -0.190(0.125) | -0.192(0.164) | -0.764***(0.202) |
| High | 0.089(0.093) | 0.198*(0.102) | 0.182**(0.069) | -0.022(0.102) | 0.050(0.132) | 0.061(0.112) |
| **Urban or rural residency** |  |  |  |  |  |  |
| Rural area (reference) | - | - | - | - | - | - |
| Urban area | 0.397***(0.078) | 0.416***(0.085) | 0.360***(0.057) | 0.205**(0.083) | 0.351**(0.109) | 0.585***(0.113) |
| **Consistency check** | 0.103***(0.026) | 0.184***(0.027) | 0.045**(0.018) | 0.109***(0.027) | 0.038(0.035) | 0.042(0.030) |
| **_Cons** | 0.009(0.263) | -0.930***(0.271) | 0.105(0.166) | 0.221(0.260) | 0.432(0.322) | -0.447(0.307) |
| **Pseudo R^2^** | 2.713 | 1.867 | 16.708 | 3.578 | 1.623 | 1.654 |

*Note: (a) Standard errors in parentheses. (b) *** p < 0.01, ** p < 0.05, * p < 0.1*

**Regression results for utilities elicited using chained TTO method**

| **Covariates** | **Profile 1** | **Profile 2** | **Profile 3** | **Profile 4** | **Profile 5** | **Profile 6** |
| --- | --- | --- | --- | --- | --- | --- |
| **Age** | 0.015***(0.003) | 0.007*(0.003) | 0.000(0.003) | 0.016***(0.002) | 0.012(0.007) | 0.005(0.006) |
| **Marital status** |  |  |  |  |  |  |
| Single/ Divorced/Widowed (reference) | - | - | - | - | - | - |
| Married/Co-habitation | 0.405*(0.174) | -0.287(0.222) | -1.069***(0.199) | 0.190(0.154) | 0.205(0.478) | 0.307(0.395) |
| **Household income** |  |  |  |  |  |  |
| <£15599/undisclosed (reference) | - | - | - | - | - | - |
| Between £15599 and £36399 | 0.259*(0.113) | 0.365**(0.143) | 0.305*(0.128) | 0.454***(0.100) | -0.381(0.309) | -0.208(0.263) |
| >£36400 | 0.235**(0.090) | 0.401**(0.114) | 0.360**(0.102) | 0.423***(0.080) | -0.338(0.246) | -0.562**(0.215) |
| **Education level** |  |  |  |  |  |  |
| GCSE grade D-G / CSE / NVQ level 1/ Foreign qualifications / other/ No formal qualifications (reference) | - | - | - | - | - | - |
| Higher education below a degree/ GCE A-Level or AS-Level / NVQ level 3/ GCSE grade A*-C / GCE O-Level / NVQ level 2 | -0.069(0.053) | 0.329***(0.068) | 0.245***(0.063) | -0.110*(0.049) | -0.173(0.146) | 0.090(0.127) |
| Degree level or above | -0.455***(0.115) | 0.392**(0.146) | 0.773***(0.133) | -0.440***(0.105) | -0.749*(0.316) | -0.113(0.300) |
| **Employment status** |  |  |  |  |  |  |
| Employed Part-Time/Unemployed (reference) | - | - | - | - | - | - |
| Retired | -0.471**(0.185) | 0.446(0.235) | 1.206***(0.210) | -0.062(0.160) | -0.964(0.508) | -0.979*(0.469) |
| Employed Full-Time/Self-Employed | -0.226(0.118) | 0.286(0.150) | 0.703***(0.134) | 0.141(0.105) | -0.152(0.323) | -0.265(0.271) |
| **Physical activity level** |  |  |  |  |  |  |
| Low (reference) | - | - | - | - | - | - |
| Median | -0.061(0.132) | 0.350*(0.167) | 0.764***(0.150) | -0.044(0.113) | -0.328(0.360) | -0.191(0.305) |
| High | 0.113(0.113) | 0.481**(0.141) | 0.636***(0.127) | 0.034(0.096) | -0.249(0.305) | 0.075(0.257) |
| **Urban or rural residency** |  |  |  |  |  |  |
| Rural area (reference) | - | - | - | - | - | - |
| Urban area | -0.292**(0.094) | 0.120(0.119) | 0.452***(0.106) | -0.215**(0.083) | -0.675**(0.257) | -0.316(0.244) |
| **Consistency check** | 0.200***(0.035) | 0.076(0.044) | -0.142**(0.040) | 0.196***(0.031) | 0.077(0.095) | 0.018(0.079) |
| **_Cons** | -0.213(0.240) | -0.922**(0.304) | -0.731**(0.272) | -0.614**(0.211) | 1.345*(0.657) | 0.789(0.567) |
| **Pseudo R^2^** | -7.807 | -11.759 | -11.554 | -15.910 | -18.898 | 2.094 |

*Note: (a) Standard errors in parentheses. (b) *** p < 0.01, ** p < 0.05, * p < 0.1*
